# Traditional Chinese Medicine Tang-Luo-Ning Ameliorates Sciatic Nerve Injuries in Streptozotocin-Induced Diabetic Rats

**DOI:** 10.1155/2013/989670

**Published:** 2013-10-28

**Authors:** Da-Wei Zou, Yan-Bin Gao, Zhi-Yao Zhu, Hui Zhou, Tao-Jing Zhang, Bu-Man Li, Jin-Yang Wang, Min-Zhou Li, Ming-Fei Ma, Na Zhang

**Affiliations:** ^1^School of Traditional Chinese Medicine, Capital Medical University, Number 10 Youanmenwai Xitoutiao, Fengtai District, Beijing 100069, China; ^2^Endocrinology Department, Oriental Hospital of Beijing University of Chinese Medicine, Beijing, China; ^3^Traditional Chinese Medicine Department, Shougang Hospital of Peking University, Beijing, China

## Abstract

Diabetic peripheral neuropathy (DPN) is a common microvascular complication of diabetes associated with high disability rate and low quality of life. Tang-Luo-Ning (TLN) is an effective traditional Chinese medicine for the treatment of DPN. To illustrate the underlying neural protection mechanisms of TLN, the effect of TLN on electrophysiology and sciatic nerve morphology was investigated in a model of streptozotocin-induced DPN, as well as the underlying mechanism. Sciatic motor nerve conduction velocity and digital sensory nerve conduction velocity were reduced in DPN and were significantly improved by TLN or **α**-lipoic acid at 10 and 20 weeks after streptozotocin injection. It was demonstrated that TLN intervention for 20 weeks significantly alleviated pathological injury as well as increased the phosphorylation of ErbB2, Erk, Bad (Ser112), and the mRNA expression of neuregulin 1 (Nrg1), GRB2-associated binding protein 1 (Gab1), and mammalian target of rapamycin (Mtor) in injured sciatic nerve. These novel therapeutic properties of TLN to promote Schwann cell survival may offer a promising alternative medicine for the patients to delay the progression of DPN. The underlying mechanism may be that TLN exerts neural protection effect after sciatic nerve injury through Nrg1/ErbB2→Erk/Bad Schwann cell survival signaling pathway.

## 1. Introduction

Diabetic peripheral neuropathy (DPN) is a common microvascular complication of diabetes and affects more than 50% of diabetic patients [1]. DPN is a progressive disease characterized by pain and paresthesia and can even develop diabetic foot or gangrene [2]. Recent studies showed that the incidence of DPN is a complex mechanism mainly including (1) hyperglycemia and metabolism disorders such as activation of polyol pathway [3], nonenzymatic glycosylation [4], and activation of protein kinase C [5]; (2) activation of mitogen-activated protein kinases (MAPK) pathways [6]; (3) oxidative stress [7]; (4) lack of neurotrophic support and vascular pathologies [8]; (5) apoptosis of Schwann cells (SCs) [9]. 

As an important neuron in peripheral nerve system, SCs have become a center for many research studies in recent years as they play a key role in myelination, neurotrophic support, homeostasis, and repair after peripheral nerve injury [10–13]. Moreover, physiological interactions and reciprocal signaling between axons and SCs play critical roles in nerve development and survival, and Nrg1 is an essential modulator of SCs during development, nerve repair, and remyelination [14]. However, conventional therapy techniques for DPN, including controlling hyperglycemia, improving microcirculation, alleviating metabolic disorder, and suppressing oxidative stress, have failed to halt functional and structural changes of peripheral nerve in both humans and experimental animals [15]. Significantly, this suggests that the injuries of SCs have been activated by hyperglycemia induced metabolic disturbance, and then SCs can act independently to further exacerbate the structural and functional injuries of sciatic nerve. The therapies above could not reverse the occurred peripheral nerve injuries such as axonal degeneration, demyelination, and cell apoptosis. Consequently, improvement of SCs survival and recovery of peripheral nerve regeneration ability are of vital importance to delay the progress of DPN.

As is well known, the incidence of DPN could be attributed to a multiple mechanisms. Traditional Chinese medicine (TCM), which has the advantage of hitting multiple targets at the same time, may be a good choice for such therapeutic agents [16]. Tang-Luo-Ning (TLN) is an effective Chinese recipe in TCM for the treatment of DPN [17, 18]. According to the theory of TCM, TLN recipe mainly contains three different traditional Chinese herbs: *Astragalus membranaceus* (Fisch.) Bge (Huangqi), *Salvia miltiorrhiza *Bge. (Danshen), and *Paeonia lactiflora *Pall. (Chishao) (detailedly shown in Section 2). This recipe functions to tonify qi promote the blood circulation and remove the blood stasis. It has been reported that *Astragalus membranaceus* could reduce the serum glucose and triglyceride level and inhibit advanced glycation end products-induced inflammation as well in the diabetic individuals [19, 20]. Moreover, Astragaloside IV, the major active component of TLN recipe, could exert protective effects against DPN in streptozotocin- (STZ-) induced diabetes in rats through several interrelated mechanisms [21]. Previously, we demonstrated that TLN exerted protective effects against the progression of DPN through several interrelated mechanisms, such as improvement of oxidative damage, inhibition of dorsal root ganglion and sciatic nerve apoptosis [22, 23] in streptozotocin-induced diabetic rats (STZ-R). However, whether TLN intervention attenuates sciatic nerve injury and promotes recovery of it through enhancing SCs survival is unknown.

In our study, due to multiple-target characteristic of Chinese herbal compound, microarray expression analysis was used to screen cell-survival related genes and pathways regulated by TLN, followed further validation by quantitative real time polymerase chain reaction (qPCR) and western blot. 

## 2. Materials and Methods

### 2.1. The TLN Extract Preparation and Quality Control

TLN recipe mainly contains the ingredients from three different traditional Chinese herbs: the dried root of *Astragalus membranaceus* (Fisch.) Bge. (Huangqi, amount used 30 g per day), the dried root and rhizome of *Salvia miltiorrhiza *Bge. (Danshen, amount used 30 g per day), and the dried root of *Paeonia lactiflora *Pall. (Chishao, amount used 20 g per day) as well. All herbs were purchased from Oriental Hospital of Beijing University of Chinese Medicine (BUCM, Beijing, China). The crude drugs were carefully authenticated and then decocted and made into particle in the Manufacturing Laboratory of Oriental Hospital of BUCM. In brief, the weight proportion of *Astragalus membranaceus* (Fisch.) Bge., *Salvia miltiorrhiza *Bge., and *Paeonia lactiflora *Pall. is 3 : 3 : 2. The herbs were extracted three times with 60% ethanol (600 mL per 100 g herbs) for 1.5 h each time. The ethanol extract was pooled and concentrated to stronger liquor with a relative density of 1.25–1.30 at 70°C. The above liquor was evaporated under reduced pressure and ground into fine powder to obtain the TLN extract. The TLN extract and excipients were mixed at a ratio of 1 : 1 with 80% ethanol to pelletize. A content of 0.7875 mg/g Astragaloside IV, which is the main active component in TLN, was detected in TLN particles by HPLC-ELSD (shown in Supplementary Figure 1, available on line at http://dx.doi.org/10.1155/2013/989670). TLN particles were suspended in the distilled water at appropriate concentration for the animal treatment. 

### 2.2. Animal Model and Drug Intervention

Male Sprague Dawley rats weighting 180 g–220 g were purchased from Vital River Laboratories (Beijing, China, certificate No. SCXK 2006–0009). The animals were housed in separate cages at constant temperature (20–22°C) and humidity (50%–67%) under a 12-hour light/dark cycle in specific pathogen free animal laboratory of BUCM. The animals had free access to standard chow diet (Vital River, Beijing, China) and sterilized drinking water during the period of this experiment. The experimental protocol was approved by the ethics committee of BUCM. All animal experiments were conducted in accordance with the NIH guide for the care and use of laboratory animals (NIH Publication No. 80-23; revised 1978). 

After one week of acclimatization, rats were given STZ (Sigma, 60 mg/kg body weight), and 72 hours later rats with fasting blood glucose more than 16.7 mmol/L were considered successfully induced for diabetes. The diabetic rats were randomly divided into three groups including STZ induced diabetic group (STZ group), Tang-Luo-Ning group (TLN group), and alpha-lipoic acid group (*α*-LA group); there was also a non-STZ induced group as control group (CTL group). Rats in intervention groups were administrated separate drug by gavage (5 mL/kg) from the next day at a dose of either 10 g/kg body weight (TLN group**)** or 20 mg/kg body weight (*α*-LA group), and the rats in CTL group or STZ group were treated at the same volume of distill water (5 mL/kg) by gavage for 20 weeks. The *α*-LA was purchased from Puritan's Pride Inc. (Oakdale, NY, USA). The batch number of the *α*-LA used in this experiment was 250597-09. Determination of the dose of TLN used in the present study was based on our previous works. After the last administration, all rats were fasted for 12 hours before experiment but allowed free access to water. The animals were anesthetized using 10% chloral hydrate (300–350 mg/kg) to get sciatic nerve. The sciatic nerve tissue from each mouse was also divided into two parts, one part was immediately frozen in liquid nitrogen for western blot, RNA extraction, and microarray, and another part was fixed with 4% glutaraldehyde for electron microscope, respectively.

### 2.3. Electrophysiological Measurements

Sciatic motor nerve conduction velocity (MNCV) and distal digital sensory nerve conduction velocity (SNCV) were measured after induction of anesthesia with 10% chloral hydrate (300–350 mg/kg) at two points (10 weeks and 20 weeks after intervention). Body temperature was maintained at 37°C with a warming pad. For sciatic measurement, the left sciatic motor conduction system was stimulated at the sciatic notch where sciatic nerve exits and at ipsilateral ankle via bipolar electrodes with a width of 0.1 ms. The threshold was determined when the compound muscle action appeared or disappeared, and we set at 1.5-fold above the threshold as stimulus intensity. The recorded bipolar electrodes were placed at the first interosseous muscle of the hind-paw, as well as reference electrode was placed 1 cm away from the recording electrode but between the stimulating and the recording electrode. Hind-limb SNCV was recorded in the digital nerve to the second toe by stimulating with a square-wave pulse duration of 0.05 ms using the smallest intensity current that resulted in a maximal amplitude response. The sensory nerve action potential was recorded behind the medial malleolus. The maximal SNCV was calculated from the latency to the onset of the initial negative deflection and the distance between stimulating and recording electrodes. The latencies of action potentials were measured as described below by physiological data recording system (MacLab/400, ADI, Australia) and dual-beam memory oscilloscope (VC-10, Nihon Kohden Corporation, Japan). Average conduction time was calculated after 7–10 measurements. Sciatic MNCV and distal digital SNCV were measured and calculated based on the method [24].

### 2.4. Morphological Observation of the Sciatic Nerve

Approximately 0.1 cm of sciatic nerve was removed from the lower edge of the left piriformis and cut into pieces about 1 mm^3^ by volume. After prefixing with cold, 4% glutaraldehyde nerve tissues were fixed with 1% osmic acid and dehydrated and embedded in 1 : 100 mixture of Epon 812 and 100% aceton. Semithin sections were cut and stained with toluidine blue before they were further cut into 50 nm ultrathin sections using an ultramicrotome (8800, LKB, Bromma, Sweden). After double staining with uranyl acetate and lead nitrate, morphological changes of the sciatic nerves were observed by transmission electron microscopy (H-600, Hitachi, Japan). Images were taken by imaging systems (Moticam 2306, Motic Instruments Inc., Canada).

### 2.5. RNA Extraction and Microarray

RNA extraction and microarray were performed according to manufacturer's protocols by Shanghai Biotechnology Co., Ltd. TRIZOL Reagent (Catalogue number 15596-018, Life technologies, Carlsbad, CA, USA) was added into sciatic nerve, and total RNA was isolated using RNeasy Kit (Qiagen). RNA integrity number (RIN) and 28 s/18 s ratio were determined by Agilent BioAnalyzer 2100. RNAs with RIN ≥ 7.0 and 28S/18S > 0.7 were deemed to be of sufficient quality. RNA concentration and A260/A280 ratio were determined by Nanodrop ND-1000. RNAs were purified by QIAGEN RNeasy Kit, followed by cDNA synthesis and cRNA fluorescent labeling, purifying, and shearing. Probes were hybridized with Rat Gene Expression Microarray slides (4 × 44 K microarray, Agilent, Catalogue number G2519F-014879) in a hybridization oven (Agilent G2545A). After thorough washing, array slides were scanned by Agilent scanner with both 100% and 10% PMT setting. Results were analyzed by Agilent software.

### 2.6. Microarray Data Analysis

With threshold of fold change between experimental samples (TLN group) and control samples (STZ group, CTL group) set at 2, genes that were differentially regulated by TLN were identified. Genes that were first downregulated >2-fold (compared with CTL) by STZ and then upregulated >2-fold (compared with STZ) by TLN were considered as upregulated genes, and the downregulated genes were opposite. Differentially regulated genes by TLN were analyzed using an online SAS system for hierarchical clustering, Gene ontology (GO) enrichment analysis and Kyoto Encyclopedia of Genes and Genomes pathway analysis (enrichment *P* < 0.05).

### 2.7. qPCR

To confirm microarray data and further screen cell survival related pathway, Nrg1, GRB2-associated binding protein 1 (Gab1), mammalian target of rapamycin (Mtor), and phosphoinositide-3-kinase, catalytic, beta polypeptide (Pik3cb) were selected for validation by qPCR. Primers were designed by Sheng Gong Biotech (Shanghai, China) using Primer Premier 5.0 software (PREMIER Biosoft International, CA, USA). The sequences of Nrg1, Pik3cb, Mtor, Gab1, and GAPDH were the following: Nrg1: forward primer 5′-TGGCACATCCATCCAAATAC-3′, reverse primer 5′-GTAGCATGCTGCTGGGTCTA-3′; Pik3cb: forward primer 5′-AGATGTTGCTCAGCTTCAGG-3′, reverse primer 5′-TT CATCACTCATCTGTCGCA-3′; Mtor: forward primer 5′-AGA ACCACATGCCACACAGT-3′, reverse primer 5′-CTTTGGCATTTGTGTCCATC-3′; Gab1: forward primer 5′-CTCCTGAGACCACAAAGCAA-3′, Reverse primer 5′-AACGCTAGCTGCTTCTCACA-3′; GAPDH: Forward primer 5′-CAACTCCCTCAAGATTGTCAGCAA-3′, Reverse primer: 5′-GGCATGGACTGTGGTCATGA-3′. RNA was reverse transcribed by M-MLV Reverse Transcriptase (CK2801A, Takara) according to manufacturer's protocols, and qPCR was performed on qPCR machine (ABI 7500, Life Technologies, USA) with the following program settings: 94°C for 15 min, 94°C for 15 s, 60°C for 34 s, and 72°C for 15 s for 40 cycles followed by 72°C for 10 min. All qPCRs were performed in duplicate, and relative expression was calculated using the 2^−ΔΔCT^ method [25].

### 2.8. Western Blot

To determine the expression level of (P-) ErbB2, extracellular signal-regulated kinase (Erk), P-Erk, protein kinase B (PKB/Akt), P-Akt, bad, and P-bad Ser112/Ser136, the sciatic nerve tissues were quickly pulverized in prechilled mortar and then fractionated by SDS-PAGE. 40 *μ*g of protein was separated by electrophoresis and transferred to Nitrocellulose membrane (Millipore, USA). Membrane was blocked in 5% nonfat dry milk in Tris-buffered saline (containing 0.1% Tween-20, PH = 7.6) for 1 h and incubated with primary antibodies at 4°C overnight. The primary antibodies were as follows: mouse monoclonal anti-Erk1/2 antibody (dilution: 1 : 3000), mouse monoclonal anti-Phospho-Erk1/2 antibody (dilution: 1 : 2000), rabbit monoclonal anti-Bad (D24A9) antibody (dilution: 1 : 2000), rabbit monoclonal anti-Phospho-Bad (Ser112) (40A9) antibody (dilution: 1 : 500), rabbit monoclonal anti-Phospho-Bad (Ser136) (D25H8) antibody (dilution: 1 : 500), rabbit monoclonal anti-Akt antibody (dilution: 1 : 3000), and rabbit monoclonal anti-Phospho-Akt (Ser473) antibody (dilution: 1 : 2000); the antibodies above were all purchased from Cell Signaling Technology (USA). Rabbit polyclonal anti-ErbB2 antibody (dilution: 1 : 4000, Novus, Biologicals, Inc.; Littleton, CO, USA), Rabbit polyclonal anti-Phospho-ErbB2 (Tyr1248) antibody (dilution: 1 : 3000, Novus, Biologicals, Inc.; Littleton, CO, USA), after washing 3 × 10 min with Tris-buffered saline, horseradish peroxidase conjugated secondary antibody (dilution 1 : 1000: P-Bad ser112/ser136, 1 : 2000: other antibodies) was added and incubated for 1 hr with shaking. Target proteins were visualized using enhanced chemiluminescence reagents and exposed to X-Ray film in dark room. Densitometry analysis was performed on protein bands using software IPP 6.0 (Media Cybernetics Inc., USA).

### 2.9. Statistical Analysis

Differential gene expression and bioinformatics analysis were performed using online SAS analysis system provided by Shanghai Biotechnology Corporation. All other data were statistically analyzed by SPSS13.0 software. Average value ± standard deviation (Mean ± SD) were calculated, one-way ANOVA (analysis of variance) and LSD-*t* test (least significant difference *t* test) for multiple samples multiple comparison were performed, and *P* < 0.05 were considered as statistically significant.

## 3. Results

### 3.1. Function and Morphology of Sciatic Nerve

#### 3.1.1. TLN Increases Nerve Conduction Velocity in STZ-Induced Diabetic Rats

**Figure 1:**
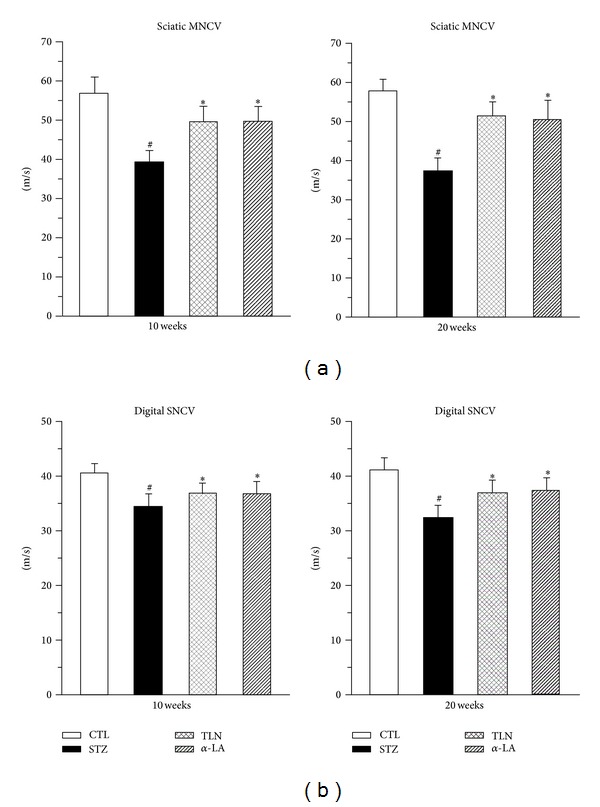
Effects of TLN and *α*-LA on (a) sciatic MNCV and (b) distal digital SNCV at 10 and 20 weeks after streptozotocin injection. *n* = 8 per group. Data are expressed as mean ± SD. ^#^ *P* < 0.05 versus CTL group, *P < 0.05 versus STZ group; sciatic MNCV and distal digital SNCV of STZ group rats with streptozotocin injection decreased compared with CTL group (*P* < 0.05). Tang-Luo-Ning and alpha-lipoic acid intervention both increased sciatic MNCV and distal digital SNCV of injured sciatic nerve compared with STZ group (*P* < 0.05). MNCV: Motor nerve conduction velocity. SNCV: sensory nerve conduction velocity. Four groups: CTL group: nonstreptozotocin-induced group, STZ group: streptozotocin-induced diabetic group, TLN group: Tang-Luo-Ning group, and *α*-LA group: alpha-lipoic acid group.

Compared with the STZ group, an obvious improvement of sciatic MNCV and distal digital SNCV were observed after 10 weeks of TLN treatment (Figure 1), and this trend was even more profound after 20 weeks of TLN treatment. Moreover, the trend of MNCV after *α*-LA treatment was consistent with published data [26]. In brief, results demonstrated that TLN intervention increased MNCV and distal digital SNCV of injured sciatic nerve and improved the lower nerve function.

#### 3.1.2. TLN Alleviates Pathological Injury of Sciatic Nerve

**Figure 2:**
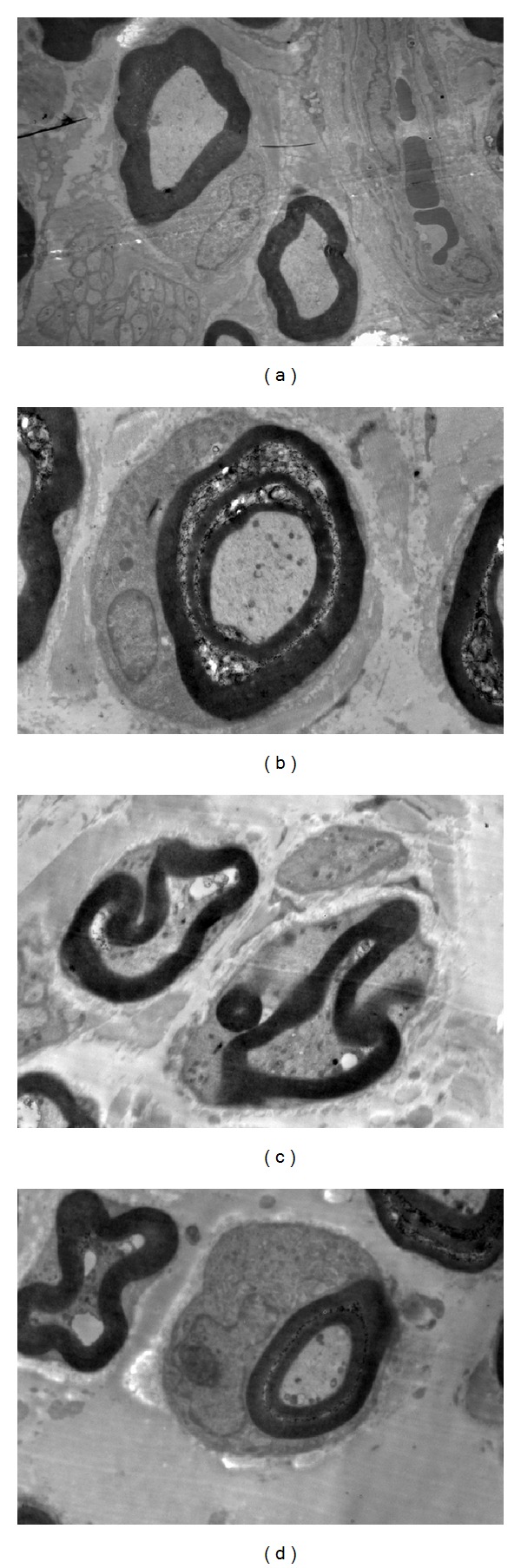
Transmission electron micrographs showing the ultrastructure of sciatic nerve fiber (×10000) (a) CTL rats: intact myelinated axon. (b) STZ rats: serious demyelination, axial degeneration, and Schwann cell proliferation. (c) TLN rats: moderate segmental demyelination and axial degeneration. (d) *α*-LA rats: moderate segmental demyelination, axial degeneration, and Schwann cell proliferation. Four groups: CTL group: nonstreptozotocin-induced group, STZ group: streptozotocin-induced diabetic group, TLN group: Tang-Luo-Ning group, and *α*-LA group: alpha-lipoic acid group.

In control rats, there were fair-arranged nerve fibers, mainly with myelinated nerve of thick myelin and large axon diameter simultaneously. In diabetic rats, serious demyelination, axial degeneration and reduced number of myelinated nerve fibers were observed on transmission electron microscope, and the pathological injury in this study corresponded to typical morphological changes of DPN. Likewise, the pathologic and morphological changes in the sciatic nerves of diabetic rats treated with TLN and *α*-LA were both greatly alleviated (Figure 2). No significant changes were observed in the number and proportion of myelinated and unmyelinated nerve fibers in TLN and *α*-LA group. The results demonstrated that TLN could be counteracting against the pathological morphology changes of sciatic nerve in STZ-induced diabetic rats.

### 3.2. Schwann Cells Survival of Sciatic Nerve

#### 3.2.1. Microarray Bioinformatics Analysis of Differentially Regulated Genes by TLN

**Figure 3:**
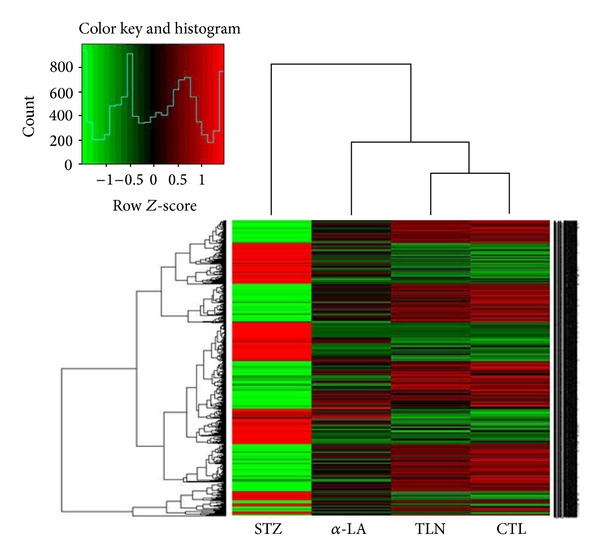
Heat map showed the differentially expressed genes regulated by TLN between four groups. Red color indicates over expressed genes, whereas green color indicates the opposite. The color scale bar is shown. Four groups: CTL group: nonstreptozotocin-induced group, STZ group: streptozotocin-induced diabetic group, TLN group: Tang-Luo-Ning group, and *α*-LA group: alpha-lipoic acid group.

**Figure 4:**
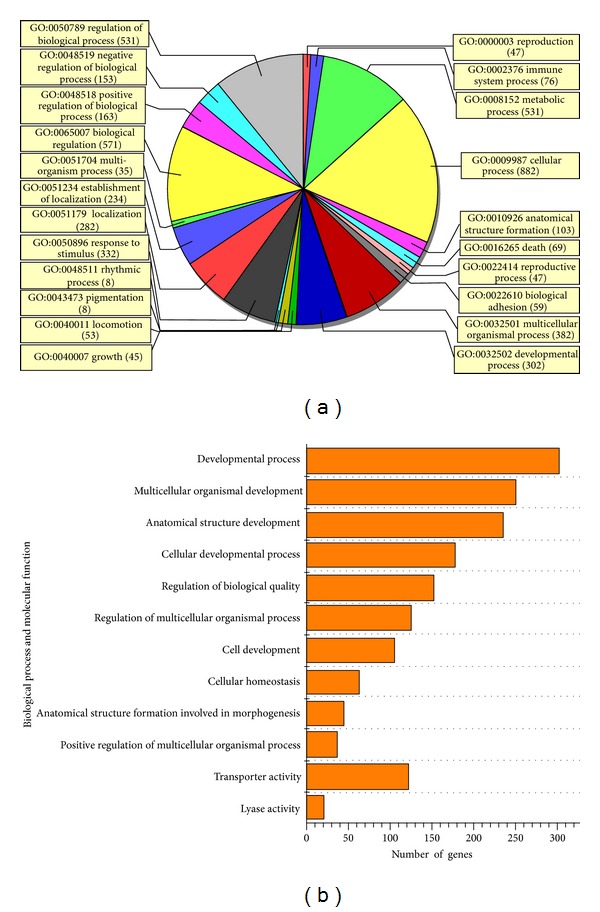
GO analysis and significantly altered categories regulated by TLN. (a) Gene ontology category of biological process for differentially expressed genes regulated by TLN. (b) The significant GO categories for differently expressed genes. The vertical axis represents the GO category, and the horizontal axis represents the number of genes changed in each category. There are 12 categories that were significantly altered (enrichment *P* value < 0.05 and number of changed genes >20). The first 10 categories are involved in biological process; the last two are involved in molecular function. GO: gene ontology.

Using an online SAS system, cluster analysis was performed and the resultant heat map allowed us to visualize the differential gene expression patterns between the experimental groups (Figure 3). GO analysis encompasses three domains: molecular function, biological process, and cellular component. GO analysis showed that the functions of genes that were differentially regulated by TLN mainly focused on cellular homeostasis, cell development, and anatomical structure formation (Figure 4). Pathway analysis showed that cell survival related pathways regulated by TLN contained ErbB signaling pathway, neurotrophin signaling pathway, and phosphatidylinositol signaling system (Table 1). These results indicated that TLN may exert protective effects against morphological changes of sciatic nerve through regulating these genes of above pathways, such as Nrg1, Pik3cb, Mtor, and Gab1 (Tables 1 and 2).

#### 3.2.2. TLN Upregulates Gene Expression of Nrg1, Mtor, and Gab1 in Sciatic Nerve of Diabetic Rats

**Figure 5:**
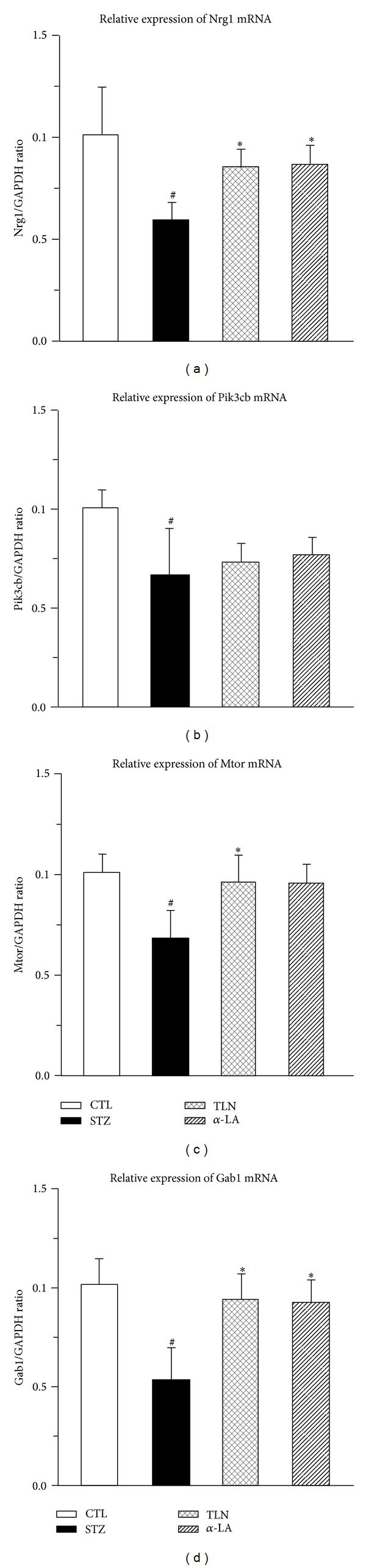
Effect of TLN and *α*-LA on cell survival related genes in sciatic nerve of streptozotocin-induced rats. Relative expression level change (2^−ΔΔCT^) of 4 genes in qPCR, (a) Nrg1, (b) Pik3cb, (c) Mtor, and (d) Gab1. Statistical analysis was performed using the ANOVA procedure *t* test (LSD). Data are expressed as mean ± SD. ^#^ *P* < 0.05, when compared with CTL group; *P < 0.05, when compared with STZ group. The mRNA expression of Nrg1, Pik3cb, Mtor, and Gab1 decreased compared with CTL group (*P* < 0.05); TLN intervention could increase the expression level of Nrg1, Mtor, and Gab1 compared with STZ group (*P* < 0.05). The alpha-lipoic acid intervention trend was consistent with Tang-Luo-Ning except Gab1. There were no significant differences of Pik3cb mRNA between the three groups (STZ, *α*-LA, and TLN). Four groups: CTL group: nonstreptozotocin-induced group, STZ group: streptozotocin-induced diabetic group, TLN group: Tang-Luo-Ning group, and *α*-LA group: alpha-lipoic acid group. ANOVA: analysis of variance; LSD-*t* test: least significant difference *t* test; qPCR: quantitative real time polymerase chain reaction.

Cell-survival genes (Nrg1, Mtor, Gab1, and Pi3kcb) identified from the microarray analysis were selected and performed qPCR for validation. Results showed that the TLN intervention upregulated the Nrg1, Mtor, and Gab1 expression levels, while the expression pattern of Pi3kcb were not consistent with the microarray data (Table 2 and Figure 5). Since Pi3kcb is the crucial gene of the Neurotrophin signaling pathway and phosphatidylinositol signaling system, there was no sense to do deeper study the two pathways.

#### 3.2.3. TLN Upregulates Expression of P-ErbB2, P-Erk, and P-Bad (Ser112) in Sciatic Nerve of Diabetic Rat

**Figure 6:**
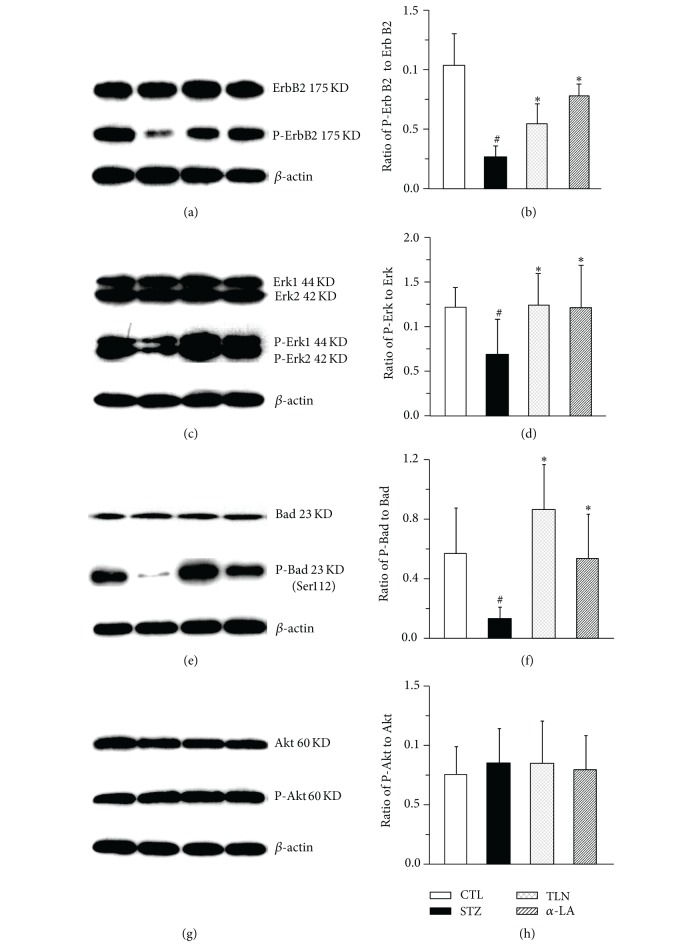
Effect of TLN and *α*-LA on Nrg1/ErbB2 downstream signal pathway in sciatic nerve of streptozotocin-induced rats. A representative western blot ((a), (c), (e), and (g)) and the grey value analyses of protein expression ((b), (d), (f), and (h)) in sciatic nerves from different experimental groups. ((a), (b)) Ratio of P-ErbB2 to T-ErbB2 (P/T); ((c), (d)) ratio of P-Erk to T-Erk (P/T); ((e), (f)) ratio of P-Bad (Ser112) to T-Bad (P/T); ((g), (h)) ratio of P-Akt to T-Akt (P/T). Statistical analysis was performed using the ANOVA procedure *t* test (LSD). Data are expressed as mean ± SD. ^#^ *P* < 0.05 versus CTL group, *P < 0.05 versus STZ group. *n* = 4 per group. The protein expression of ratio of ErbB2 Erk, Bad (P/T) decreased compared with CTL group (*P* < 0.05), while Tang-Luo-Ning and alpha-lipoic acid intervention could increase the ratio above compared with STZ group (*P* < 0.05). There were no significant differences of ratio of Akt (P/T) between the four groups. Four groups: CTL group: nonstreptozotocin-induced group, STZ group: streptozotocin-induced diabetic group, TLN group: Tang-Luo-Ning group, and *α*-LA group: alpha-lipoic acid group. ANOVA: analysis of variance; LSD-*t* test: least significant difference *t* test.

In brief, we focused on Nrg1/ErbB2 pathway and do further validation by western blot. It is demonstrated that there was little variation of total ErbB2, Erk, Bad, Akt, and P-Akt after STZ injection (*P* > 0.05), and the phosphor-Bad (Ser136) cannot be detected by western blot. Likewise, phosphorylated levels of ErbB2, Erk, and Bad (Ser112) declined 3.6-fold, 1.9-fold, 4.4-fold in the STZ group (no intervention after STZ injection) when compared with the control group, respectively. In contrast, the expression levels of phosphorylated protein were similar to normal rat with TLN and *α*-LA intervention after STZ injection (Figure 6). 

Taken together, these results from western blot and qPCR demonstrated that TLN could enhance NRG/ErbB2→Erk/Bad dependent SCs-survival signal pathway (Figures 5 and 6). 

## 4. Discussion

The present study provides direct evidence for a crucial role of TLN in function and morphology of sciatic nerve after diabetes. The protective effects of the compound recipe TLN may be attributed to promote SCs-survival through the activation of Nrg1/ErbB2→Erk/Bad (Ser112) signal pathway by increasing the phosphorylation of ErbB2, Erk, and Bad (Ser112) at a protein level and mRNA expression of Nrg1, Mtor, and Gab1. This finding supports the hypothesis that TLN was a critical regulator in SCs survival and recovery of peripheral nerve regeneration ability. Recently there has been a great deal of interest in SCs due to the key role of it in myelination regeneration and repair after peripheral nerve injury. 

DPN is a long-term common complication of diabetes. In this study, STZ was used to induce diabetic neuropathy model, which was widely performed in Sprague—Dawley rats [21], Wistar rats [27], and transgenic mice [28]. The STZ-induced diabetic rats beard the typical characteristics of DPN including demyelination and axial degeneration of sciatic nerve and reduction of peripheral nerve conduction velocity, which were consistent with published observations [29]. In addition, *α*-LA was selected as a positive control in the present study because of its antioxidative activity [30, 31].

In fact, ErbB2 signal pathway was most closely linked to SCs survival among the three screened signal pathways by microarray (Table 1), and Nrg1, Mtor, and Gab1 are key genes of ErbB2 signal pathway. Mtor is a conserved serine/threonine protein kinase and a member of the phosphatidylinositol 3-kinase-related kinase family. Mtor plays key roles in sensing nutrition signal, regulation of cell growth and proliferation [32], and regulation of insulin response by the insulin response element [33]. Meanwhile, due to the proapoptosis effect of c-Jun-NH2-terminal kinase pathway activation, Gab1 has an indirect antiapoptosis effect. Biochemical analysis suggested Gab1 played a positive role in coupling the signal relay between cytokine receptors and the Erk pathway [34]. More important, Nrg1, also known as glial growth factor, is mainly produced by SCs and transported by axons and plays a crucial role in SCs survival. Nrg1 belongs to Neuregulin gene family which contains four members termed as Nrg1, Nrg2, Nrg3, and Nrg4. Among them, Nrg1 is the most characteristic gene, and it is believed to be able to rescue SCs from apoptosis and act as a predominant survival factor during SCs maturation [35]. Recently studies also demonstrated that NRG signal provided trophic support to myelin-producing cells and contributed to myelination in peripheral nerve system and velocity of conduction [36, 37]. In our study, these three genes discussed above appeared to be key factors through which TLN might promote SCs survival.

The binding and activation of Nrg1 and ErbB2/ErbB3 coreceptor to activate the downstream signal pathways is very important to transmit SCs survival signal. Being a family member of polypeptide growth/differentiation factors containing an epidermal growth factor-like motif, Nrg1 could activate membrane-associated ErbB tyrosine kinase receptors and mediate SCs differentiation, proliferation, and migration through binding and activation of a heterodimeric ErbB2/ErbB3 coreceptor [38]. ErbB2 itself does not bind to NRG, while ErbB3 alone could bind to NRG but could not transmit signals. Since it is necessary for ErbB2 and ErbB3 to form a high affinity coreceptor complex to transmit SCs survival signal, the expression level of P-ErbB2 could reflect NRG receptor downstream signal intensity. In this study, the expression level of P-ErbB2 decreased after STZ induction, suggesting that there was a close relationship between Nrg1-ErbB2 signal transduction and sciatic nerve morphology change/conduction velocity. These data contradict with some reports [39] but agree with some others [40–42]. The underlying causes for this discrepancy are unknown. The present study also showed that TLN enhanced NRG receptor signal intensity through increasing Nrg1 mRNA expression and phosphorylation of ErbB2 at a protein level after peripheral nerve injury in STZ-R.

In addition, after binding to the high-affinity ErbB2/ErbB3 coreceptor complex, Nrg could potentially activate several distinct signaling pathways, mainly including p21Ras/Raf-1/MEK/Erk cascade [43] and PI3K/Akt signaling pathway [44]. The phosphorylation of bad either on serine 112 by Erk or serine 136 by Akt promotes its dissociation from the cytosolic protein 14-3-3, leading to the release of Bcl-2 and Bcl-xl to inhibit SCs apoptosis. Obviously, the phosphorylation of bad is the key link for TLN to promote SCs survival. One study showed that NRG-ErbB2 receptor signaling promoted SCs survival through a downstream PI3K/Akt/Bad pathway [41]. In this study, we detected that the significant change of phospho-Erk and phospho-Bad (Ser112), accompanying insignificant variation of phospho-Akt and no detection of phospho-Bad at the ser 136 residue, suggesting that SCs-survival effect by TLN could be due to the Erk/Bad (Ser112) signal but not the PI3K/Akt/Bad (Ser136) signal.

Despite a marked improvement in the function and morphology of sciatic nerve with TLN intervention after STZ injection, administration of TLN did not completely halt the progression of sciatic nerve injury. This suggests that there may be other signaling pathways involved in pathogenesis of nerve damage. Furthermore, although the Nrg1/ErbB2 receptor dependent SCs-survival effect of TLN is explicit in this *in vivo *animal model, its biologic relevance *in vitro* remains unclear. It will be important to evaluate the therapeutic role of TLN in future *in vitro *studies. 

## 5. Conclusion

In conclusion, the findings of the current study provide support to the hypothesis that compound recipe TLN could inhibit SC apoptosis, which lead to an improvement in sciatic MNCV, distal digital SNCV and sciatic nerve pathological injury, suggesting TLN offering a promising alternative medicine for the DPN patients to delay the progression. The mechanism of the protective effect of TLN on promoting SCs survival may be partly due to activate Nrg1/ErbB2→Erk/bad signal pathway.

## Supplementary Material

Tang-Luo-Ning (TLN), which mainly contains the ingredients from three different traditional Chinese herbs, was used in the experiment. A content of 0.7875 mg/g Astragaloside IV, which is the main active component of TLN, was detected in TLN particles by HPLC-ELSD.

## Figures and Tables

**Table 1:** Cell survival-related pathways regulated by TLN.

Pathway name	Positive and negative genes	Enrichment test *P* value
ErbB signaling pathway	Nrg1 Pik3cb Gab1 Mtor	0.0121
Neurotrophin signaling pathway	Pik3cb Gab1	5.0*E* − 4
Phosphatidylinositol signaling system	Pik3cb	0.0057

Enrichment test *P* value (*P* < 0.05) means significant. Some genes listed are involved in more than one pathway. Nrg1: Neuregulin 1; Mtor: Mammalian target of rapamycin; Gab1: GRB2-associated binding protein 1.

**Table 2:** Major genes in cell survival-related pathways upregulated by TLN.

Probe Id	Gene Id	Gene symbol	Gene name	FC: STZ versus CTL	FC: TLN versus STZ	FC: *α*-LA versus STZ
A_44_P438143	112400	Nrg1	Neuregulin 1	0.3638	2.2264	2.1083
A_43_P12820	85243	Pik3cb	Phosphoinositide-3-kinase, catalytic, and beta polypeptide	0.3298	3.3562	2.8317
A_44_P191924	56718	Mtor	Mechanistic target of rapamycin (serine/threonine kinase)	0.3786	2.2988	1.9853
A_44_P176342	361388	Gab1	GRB2-associated binding protein 1	0.2205	4.8388	3.8136

FC: fold change.
