# Supplementary material for: Traditional Chinese Medicine Tang-Luo-Ning Ameliorates Sciatic Nerve Injuries in Streptozotocin-Induced Diabetic Rats
Source: Evid Based Complement Alternat Med. 2013 Oct 28;2013:989670. doi: 10.1155/2013/989670 (PMC3830865; doi:10.1155/2013/989670)

Supplemental material

Figure 1. High-performance liquid chromatogram of Astragaloside Ⅳ in Tang-Luo-Ning particles

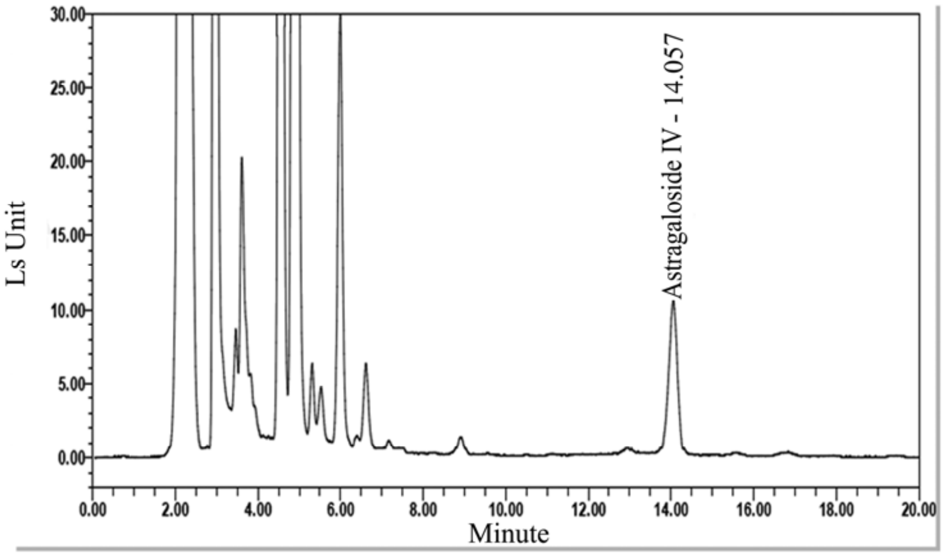

Supplement: Supplementary file 1 — Tang-Luo-Ning (TLN), which mainly contains the ingredients from three different traditional Chinese herbs, was used in the experiment. A content of 0.7875 mg/g Astragaloside IV, which is the main active component of TLN, was detected in TLN particles by HPLC-ELSD. [file 989670.f1.pdf]
